# Clinical Significance of Baseline Neutrophil-to-Lymphocyte Ratio in Patients With Ischemic Stroke or Hemorrhagic Stroke: An Updated Meta-Analysis

**DOI:** 10.3389/fneur.2019.01032

**Published:** 2019-10-04

**Authors:** Si-Ying Song, Xiao-Xi Zhao, Gary Rajah, Chang Hua, Rui-jun Kang, Yi-peng Han, Yu-chuan Ding, Ran Meng

**Affiliations:** ^1^Department of Neurology, Xuanwu Hospital, Capital Medical University, Beijing, China; ^2^Advanced Center of Stroke, Beijing Institute for Brain Disorders, Beijing, China; ^3^Department of China-America Institute of Neuroscience, Xuanwu Hospital, Capital Medical University, Beijing, China; ^4^Department of Neurosurgery, Wayne State University School of Medicine, Detroit, MI, United States; ^5^Department of Neurosurgery, Jacobs School of Medicine and Biomedical Sciences, University at Buffalo, Buffalo, NY, United States; ^6^Department of Neurosurgery, Gates Vascular Institute at Kaleida Health, Buffalo, NY, United States; ^7^Department of Cardiology, Anzhen Hospital, Capital Medical University, Beijing, China; ^8^Department of Ultrasonography, Tiantan Hospital, Capital Medical University, Beijing, China; ^9^School of Basic Medical Sciences, Capital Medical University, Beijing, China

**Keywords:** neutrophil-to-lymphocyte ratio, stroke, mortality, functional outcome, meta-analysis

## Abstract

**Background and purpose:** Stroke is a leading cause of death and acquired disability in adults today. Inflammation plays an important role in the pathophysiology of stroke. The peripheral neutrophil-to-lymphocyte ratio (NLR) is an important global inflammatory indicator becoming more mainstream in stroke care. This meta-analysis aims to evaluate the relationship between the baseline NLR and acute ischemic and hemorrhagic stroke, as well as define the clinical significance of NLR in subtypes of ischemic stroke.

**Methods:** This meta-analysis was registered in PROSPERO with the number CRD42018105305. We went through relevant articles from PubMed Central (PMC) and EMBASE. Prospective and retrospective studies were included if related to baseline NLR levels prior to treatment in patients with ischemic or hemorrhagic stroke. Studies were identified up until April 2019. The cutoff value for NLR and the sources of odds ratios (ORs)/risk ratios (RRs) were measured. Modified Rankin Scale (mRS) was used to investigate the outcomes during clinical follow-up. Predefined criteria were used to evaluate the risk of bias in eligible studies. *P*-values < 0.05 were considered statistically significant. STATA version 14.0 (STATA, College Station, TX) was used in all statistical analyses.

**Results:** Thirty-seven studies with 43,979 individuals were included in the final analysis. Higher NLR levels were correlated with increased risk of ischemic stroke (ORs/RRs = 1.609; 95% CI = 1.283–2.019), unfavorable functional outcome at 3 months (ORs/RRs = 1.851; 95% CI = 1.325–2.584), and increased mortality in patients with ischemic stroke (ORs/RRs = 1.068; 95% CI = 1.027–1.111). While in terms of hemorrhagic stroke (including SAH and ICH), elevated NLR levels only had deleterious effects on mortality (ORs/RRs = 1.080; 95% CI = 1.018–1.146).

**Conclusions:** Baseline NLR level is a promising predictor of the clinical outcomes in both ischemic and hemorrhagic stroke. In addition, elevated NLR is also associated with a high risk of ischemic stroke occurrence. However, future studies are needed to demonstrate the underlying mechanisms and further explain this association.

## Background

Stroke is a leading cause of death and acquired disability in adults (1). The major subtypes of stroke are ischemic stroke and hemorrhagic stroke, representing approximately 80% and 20% of types, respectively (2). In recent years, inflammation has been shown to have a strong relationship with the occurrence of stroke, and negative effects in both experimental and clinical data (3, 4). The inflammatory process is mediated by numerous inflammatory mediators including adhesion molecule (e.g., P-selectin), cytokines (e.g., IL-1, IL-6), chemokine (e.g., CCL2), and protease (e.g., matrix metalloproteinase-9). Furthermore, all brain cells (such as glial cells, endothelial cells, and neurons) and peripheral immune cells (such as neutrophils and lymphocytes) are contributors to the post-stroke inflammation (5, 6).

Neutrophil to lymphocyte ratio (NLR) as a reflection of innate (neutrophilic) and adaptive (lymphocytic) immune responses have been widely studied due to their convenience to obtain from peripheral blood. The increased NLR level with neutrophilic elevation and lymphocytic depletion indicates the imbalanced interaction between stroke-induced central inflammation and peripheral inflammation. Numerous studies have demonstrated that baseline NLR levels are higher in cohorts of ischemic stroke (7, 8) than hemorrhagic stroke (9, 10). Furthermore, it is suggested that higher NLR levels are correlated with poor outcomes and stroke occurrence (11–13). Several meta-analyses have indicated that increased NLR is a negative prognostic indicator in acute ischemic stroke (AIS) and spontaneous intra-cerebral hemorrhage (ICH) (14–16). Isolated analysis of ischemic and hemorrhagic stroke has created limitations in result interpretation. However, despite the different symptomology between these two subtypes of stroke, a similar pathological inflammatory pathway remains. Whether there is difference between ischemic stroke and hemorrhagic stroke with regard to prognostic value of NLR is still unclear. Elucidation of the clinical significance of NLR is needed to further explore the prognostic potential of this biomarker and its conveyed relative risk, such that it can be followed for treatment response. Our aim was to conduct a comprehensive evaluation of the relationship between baseline NLR and stroke, followed by a comparison of the prognostic value of NLR in the two main subtypes of stroke.

## Methods

### Search Strategy

This meta-analysis was registered in PROSPERO with the number CRD42018105305. Databases PubMed Central (PMC) and EMBASE were searched to identify studies for inclusion through April 2019. We used Medical subject headings and Emtree headings combined with the following keywords: “neutrophil to lymphocyte ratio OR NLR OR neutrophil OR lymphocyte” and “prognosis OR prognostic OR survival OR outcome” and “stroke OR Brain Ischemia OR Brain Infarction OR cerebral infarction OR intra-cerebral hemorrhage OR intracranial hemorrhage.” The full search strategy is presented in Supplementary Table 1.

### Study Selection

We included both prospective and retrospective studies that evaluated baseline NLR levels prior to any treatment in patients with definitive diagnosis of ischemic or hemorrhagic stroke. Eligible studies were selected if they provided an odds ratio (OR) or risk ratio (RR) with 95% confidence interval (CI) for clinical outcomes or risk of stroke incidence, or enough data to calculate these quantities. Exclusion was made if the population of study was complicated with autoimmune disorders (e.g., inflammatory bowel, primary or secondary vasculitis, rheumatoid arthritis, or anti-phospholipid syndrome) and systematic inflammatory disorders (e.g., malignancy, end stage liver disease or renal disease, or recent infection). Conference abstracts, review articles, case reports, letters, animal studies, or *in vitro* studies were not eligible for our analysis. Studies with duplicate or overlapping data were also excluded. Two reviewers (SY-S and XX-Z) independently performed the study selection and resolved any disagreements via discussion.

### Data Extraction

Data from all included studies were extracted by one author (SY-S) and was cross-checked by another author (XX-Z). The data were extracted using the name of the first author, year of publication, country, study characteristics (sample size, age, and gender), clinical characteristics (the type and subtype, severity, time of onset, comorbid status, and initial therapy of the stroke), sample time, and statistical methods used. Moreover, female-to-male gender ratio (F/M gender ratio) was calculated to precisely assess the various gender distributions among the included cohorts, which ranged from 0 to 1.8. The F/M ratio of a female-dominant composition was more than 1.2, whereas that of male-dominant cohorts was <0.8. The definition of limit interval was based on average population size in the following subgroup analysis. ORs/RRs and 95% CIs were extracted for mortality (short term or long term), functional outcome, risk of stroke incidence, and risk of post-ischemic stroke complication incidence (symptomatic intracranial hemorrhage or parenchymal hematoma). We used SPSS 19.0 to calculate RRs and 95% CIs based on the available data in studies if we received no response from the investigators after two requests. All disagreements were resolved by consensus.

### Outcomes

Outcomes were measured by the modified Rankin Scale (mRS) during clinical follow-up. Death was defined as an mRS of 6 points while unfavorable functional outcome was identified as an mRS of 3–6 points.

### Statistical Analyses

STATA version 14.0 (STATA, College Station, TX) was utilized in all analyses. Multivariate-adjusted ORs/RRs were used when possible, and univariate ORs/RRs were included in the meta-analysis if multivariate-adjusted ORs/RRs were missing. Pooled estimates with 95% CIs were derived using the Mantel-Haenszel method. We assumed that an OR is a good approximation to RR in our study due to large sample size; therefore, we pooled ORs and RRs together and simplified the description as ORs/RRs. Furthermore, we explored heterogeneity comprehensively through subgroup analysis and sensitivity analysis. Heterogeneity was assessed using the χ^2^ test and expressed as the *I*^2^ index (25% = low, 50% = medium, 75% = high) (17). When heterogeneity was more than 50%, random effects model was conducted. Assessment of publication bias was done by visual inspection of funnel plots, combined with Begg's test and Egger's test (18, 19). In addition, we applied Duval and Tweede's trim and fill method to estimate corrected effect size after adjustment for publication bias (20). Predefined criteria were used to evaluate the quality of eligible studies (21, 22). *P*-values < 0.05 were considered statistically significant.

## Results

### Study Characteristics

**Figure 1:**
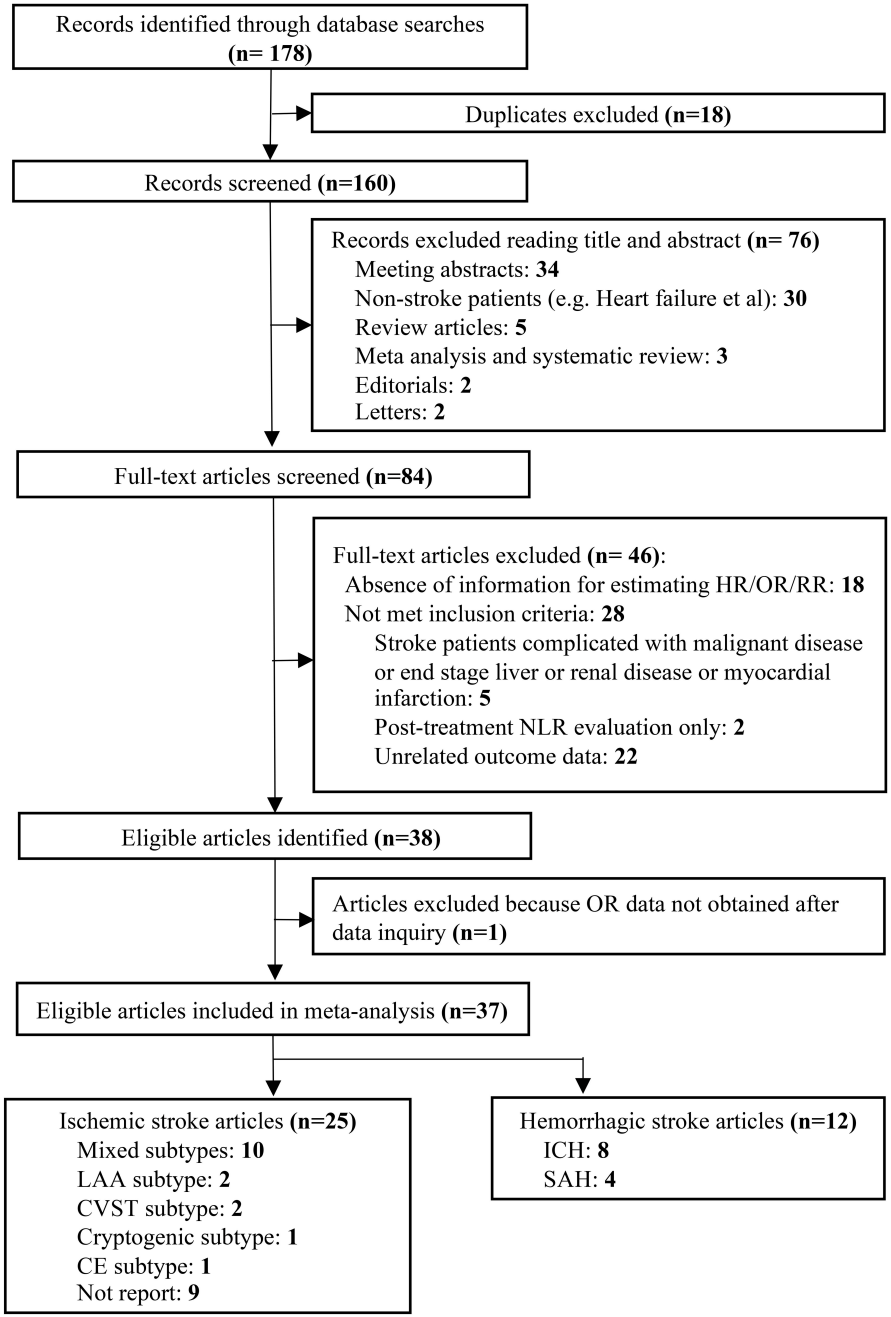
Flow diagram of the study selection process.

Our literature search identified 178 potentially relevant records. Eighteen duplicates were removed and then a total of 160 articles were screened by titles and abstracts. Seventy-six studies with irrelevant content were excluded. Furthermore, we reviewed the remaining 84 articles with full texts. In sum, 37 studies with 43,979 patients were finally included in our analysis according to the inclusion and exclusion criteria (Figure 1).

The characteristics of the included studies are shown in Table 1 (7–10, 12, 13, 23–53). Mortality, functional outcome, risks of ischemic stroke, and post-stroke complication were reported in 20, 17, 7, and 2 articles, respectively. For ischemic stroke, 25 studies included populations with AIS. The majority of studies enrolled patients with mixed stroke subtypes (*n* = 10), including large artery atherosclerosis (LAA) type, cardioembolism (CE) type, small vessel occlusion (SVC) type, cryptogenic type, and cerebral venous sinus thrombosis (CVST) type. However, several studies only evaluated specific subtypes of AIS, which were LAA subtype (*n* = 2), CVST subtype (*n* = 2), CE subtype (*n* = 1), and cryptogenic subtype (*n* = 1). For hemorrhagic stroke, a total of 12 studies reported clinical outcomes. The most frequently evaluated subtype of hemorrhagic stroke was ICH (*n* = 8) and subarachnoid hemorrhage (*n* = 4). In terms of comorbid status, a large number of studies evaluated the presence of hypertension (*n* = 33), diabetes mellitus (DM) (*n* = 31), and hyperlipidemia (*n* = 21) in their populations. Fifteen articles reported the presence of vascular disease. Current smoking status was described in 25 studies. Initial stroke therapy included antiplatelet (*n* = 14), anticoagulation (*n* = 11), thrombolysis (*n* = 4), and mechanical thrombectomy (*n* = 1). Blood samples were mostly drawn on admission (*n* = 14) or in the first 24 h after admission (*n* = 15). Four different methods for defining cutoff values were observed in the included studies. Region under the curve (ROC) analysis was used most frequently (*n* = 28), followed by the continuous (*n* = 11) and 4th quartiles (*n* = 3). Cutoff values of NLR varied between studies, ranging from 2.1 to 14, with respect to demographic characteristics among the cohorts, such as age, gender, and country of origin. Sixteen studies enrolled elderly population, the median or mean age of whom was >65 years. More than 50% of the included cohorts were with male dominant composition (*n* = 22). The number of cohorts originally from Eastern countries (*n* = 21) was nearly equal to that of cohorts from Western countries (*n* = 16). Twenty-one studies had quality scores more than 7, while the remaining 16 studies had scores ≤7 (Supplementary Table 3).

**Table 1:** Main characteristics of 37 eligible studies included in the meta-analysis.

**Study**	**Country**	**Study size**	**Age^**a**^**	**Gender** **(F/M)**	**Stroke^**b**^** **severity**	**Stroke type^**c**^**	**Onset time^**d**^**	**CAD**	**HBP**	**DM**	**Smoking**	**Hyperlipidemia**	**Initial stroke therapy^**e**^**	**Sample time^**f**^**	**Cutoff definition**	**Cutoff** **value**	**Outcome** **source**
Park et al. (23)	Korea	371	NR	151/220	NR	AIS-mixed subtypes*	Within 48 h	NR	69.3%	33.2%	46.6%	13.2%	Thrombolysis-8.1%	Within 48 h	NR	2.77	MV
Tokgoz et al. (24)	Turkey	255	69.37 ± 13.96	130/125	NR	AIS-mixed subtypes	Within 24 h	24.2%	58.0%	28.6%	27.3%	38.2%	Anticoagulation-10.0%; Antiplatelet-29.7%	On admission	Median	5	MV
Akil et al. (25)	Turkey	38	50.5 ± 13.9	15/23	NR	AIS-LAA subtype*	NR	Non	Non	Non	26.3%	Non	NR	Within 48 h	ROC	2.5	MV
Brooks et al. (7)	USA	116	68 (18–93)	37/31	NIHSS: 17 (1–48)	AIS	NR	NR	NR	NR	NR	NR	Thrombolysis-99.2%; MT-63.8%	On admission	ROC	3.2; 5.9	MV
Gao et al. (26)	China	60	54 ± 9	29/31	GCS: 3.40 ± 1.45	AIS	NR	NR	66.7%	51.7%	43.3%	NR	NR	NR	ROC	3.02	UV
Tokgoz et al. (27)	Turkey	151	69.37 ± 13.96	70/81	NR	AIS-LAA and CE subtype	Within 24 h	22.0%	52.9%	30.5%	29.3%	21.0%	Anticoagulation-3.3%; Antiplatelet-16.8%	On admission	ROC	4.81	MV
Maestrini et al. (28)	France and Finland	846	71 (60–80)	416/430	NIHSS: 10 (6–16)	AIS	Within 24 h	10.8%	61.3%	15.2%	NR	42.2%	Anticoagulation-6.0%; Antiplatelet-37.4%	On admission	Continuous variable; ROC	Non; 4.8	MV
Saliba et al. (8)	Israel	32,912	73.2 ± 13.6	16,980/15,932	NR	AIS-CE subtype	NR	49.7%	74.7%	32.8%	NR	NR	Non	NR	4th quartiles; Continuous variable	3.15; Non	MV
Zhao (29)	China	635	60.2 ± 1.3	185/450	NIHSS: 4 (2–7)	AIS-mixed subtypes*	Within 24 h	NR	63.3%	21.3%	37.8%	5.5%	NR	Within 72 h	ROC	2.59	UV
Guo et al. (30)	China	189	65.0 ± 10.6	66/123	NIHSS: 12 (6–16)	AIS	Within 24 h	12.2%	64.6%	30.2%	32.3%	45.0%	Antiplatelet-100%	Within 24 h	Continuous variable; ROC	Non; 10.59	MV
Kim et al. (31)	Korea	340	67.0 ± 12.3	111/229	NIHSS: 4.1 ± 4.7	AIS	NR	7.6%	66.2%	32.9%	40.0%	24.7%	NR	On admission	ROC	2.135	MV
Köklü et al. (32)	Turkey	254	(60–76)	75/179	NR	AIS-LAA subtype	NR	70.9%	76.8%	42.5%	32.8%	68.5%	Antiplatelet-100%	NR	ROC	2.6	MV
Lattanzi et al. (33)	Italy	177	67.1 ± 12.51	114/63	NIHSS: 9 (6–14)	ASICH	Within 24 h	13.0%	65.5%	22.0%	33.9%	20.3%	Anticoagulation-10.2%; Antiplatelet-20.9%	Within 24 h	ROC	4.58	MV
Wang et al. (34)	China	224	67.97 ± 13.75	83/141	GCS: 12.64 ± 3.49	ASICH	Within 24 h	NR	74.1%	8.5%	NR	NR	NR	Within 24 h	ROC	7.35	MV
Tao et al. (12)	China	336	58.5 ± 13.0	120/216	GCS: 11 (7–13)	SAH	Within 24 h	NR	56.3%	3.0%	24.1%	NR	Antiplatelet-5.1%	Within 24 h	ROC	6.28; 6.62	MV
Akboga et al. (35)	Turkey	80	42.1 ± 12.9	53/27	NR	AIS-CVST subtype	NR	NR	NR	NR	NR	NR	NR	On admission	ROC	2.1	MV
Fan et al. (36)	China	362	63 (52–76)	146/216	NIHSS: 9 (5–13)	AIS	Within 48 h	13.0%	80.7%	13.8%	NR	17.4%	NR	On admission	Continuous variable	Non	MV
Fang et al. (37)	Taiwan	1,731	NR	631/1,092	NR	AIS-mixed subtype	Within 48 h	5.2%	74.7%	40.7%	25.6%	NR	NR	Within 48 h	ROC	3.2	MV/UV
Giede-Jeppe et al. (38)	Germany	855	NR	397/458	NR	ASICH	NR	NR	81.9%	26.3%	32.2%	32.6%	Antiplatelet-31.5%	On admission	4th quartiles	2.606	MV
Huang et al. (39)	China	274	59 ± 16	164/110	NR	SAH	NR	NR	46.7%	11.8%	NR	NR	NR	NR	Continuous variable	Non	MV
Lattanzi et al. (9)	Italy	192	66.9 ± 12.5	69/123	NIHSS: 9 (6–14)	ASICH	Within 24 h	12.0%	64.1%	20.8%	20.3%	33.9%	Anticoagulation-9.4%; Antiplatelet-20.8%	Within 24 h	ROC	5.46	MV
Qun et al. (40)	China	143	70 (median)	63/80	NIHSS: 6 (5)	AIS	Within 24 h	NR	69.2%	21.0%	13.3%	NR	NR	On admission	ROC	2.995	MV
Sun et al. (41)	China	352	64.2 ± 13.8	118/234	NR	AICH	Within 24 h	NR	82.4%	12.2%	18.2%	NR	NR	Within 24 h	4th quartiles	7.85	MV
Tao et al. (42)	China	247	55.9 ± 11.9	159/88	NR	SAH	Within 24 h	NR	38.1%	10.1%	20.6%	NR	NR	Within 24 h	ROC; Continuous variable	14; Non	MV
Xue et al. (43)	China	292	61.8 ± 10.2	107/185	NR	AIS-mixed subtypes*	More than 48 h	NR	79.6%	34.6%	39.3%	30.0%	Anticoagulation-6.8%; Antiplatelet-92.1%	Within 24 h	ROC; Continuous variable	2.39; Non	MV
Yilmaz et al. (44)	Turkey	106	54.0 (14.5–99.3)	53/53	NR	AIS	NR	NR	6.6%	NR	NR	0.9%	NR	Within 24 h	ROC		UV
Zhai et al. (45)	China	307	63 ± 13	80/227	NR	AIS-mixed subtypes*	More than 48 h	NR	75.9%	35.5%	29.6%	25.7%	NR	Within 24 h	ROC	2.84	UV
Lattanzi et al. (46)	Italy	208	66.7 ± 12.4	76/132	NIHSS: 9 (6–14)	ASICH	Within 24 h	11.5%	65.5%	21.2%	20.1%	33.2%	Anticoagulation-8.7%; Antiplatelet-20.2%	Within 24 h	ROC	NR	MV
Wang et al. (10)	China	181	65.8 ± 14.3	69/112	GCS: 11.5 ± 4.2	AICH	Within 24 h	NR	86.2%	23.8%	NR	NR	NR	Within 24 h	ROC	7.35	MV
Nam et al. (47)	Korea	85	68 (mean)	37/48	NR	AIS-SUC subtype*	More than 48 h	NR	55.3%	21.3%	32.9%	20.0%	Anticoagulation-55.3%; Antiplatelet-35.3%; Both-7.1%; Thrombolysis-14.1%;	Within 72 h	Continuous variable	Non	MV
Shi et al. (48)	China	372	NR	130/242	NR	AIS-mixed subtypes*	Within 24 h	NR	77.4%	20.7%	39.2%	43.8%	NR	On admission	ROC; continuous variable	NR	MV
Yu et al. (13)	Australia	454	70.0 ± 16.0	201/253	NR	AIS	Within 24 h	NR	56.4%	19.8%	11.9%	25.6%	Anticoagulation-11.2%; Antiplatelet-30.2%	On admission	NR	4.12	MV
Kocaturk et al. (49)	Turkey	103	67 (55–74)	50/57	NIHSS: 10 (10–15)	AIS-mixed subtypes*	Within 24 h	NR	62.6%	30.8%	NR	35.5%	Thrombolysis-21.5%; Anticoagulation-6.5%; Antiplatelet-23.4%	Within 24 h	ROC	4.7	MV
Lim et al. (50)	Korea	104	NR	45/59	NR	AIS-mixed subtypes	Within 24 h	CAD-12.5%	69.2%	30.8%	24.0%	13.5%	NR	On admission	ROC	4.0506	MV
Wang et al. (51)	China	95	38.93 ± 13.53	57/38	NR	AIS-CVST subtype	NR	NR	NR	NR	NR	NR	Anticoagulation-100%	Within 24 h	ROC	4.205	MV
Giede-Jeppe et al. (52)	Germany	319	NR	221/98	GSC (3–15)	SAH	Within 24 h	NR	57.4%	NR	NR	NR	NR	On admission	ROC; continuous variable	7.05; Non	MV
Qin et al. (53)	China	213	50 (46–55)	56/157	NIHSS: 10 (5–12.3); GSC: 13 (7–15)	AICH	Within 24 h	CAD-4.2%	72.8%	9.4%	33.8%	NR	NR	On admission	Continuous variable	Non	MV

AIS, acute ischemic stroke; LAA, large artery atherosclerosis; CE, cardioembolism; SVO (lacunar), small vessel occlusion; SUC (cryptogenic), stroke of undetermined cause; CVST, cerebral venous sinus thrombosis; ICH, intracerebral hemorrhage; SAH, subarachnoid hemorrhage; ASICH, acute spontaneous intracerebral hemorrhage; CAD, coronary artery disease; HBP, high blood pressure; DM, diabetes mellitus; MT, mechanical thrombectomy; MV, multivariable model; UV, univariable model; NLR, neutrophil lymphocyte ratio; mRS, modified Rankin Scale; GCS, Glasgow Coma Score; NIHSS, National Institutes of Health Stroke Scale; NR, not reported.

a Age reported as either mean ± standard deviation or median (range), if not otherwise specified.

b Average initial stroke severity reported as mean ± standard deviation or median (range) or median (IQR). Glasgow Coma Score (GCS) or National Institutes of Health Stroke Scale (NIHSS) score was used for evaluation.

c Ischemic stroke further classified by different etiologies. Mixed type meant population had more than two subtypes of AIS. Studies with “*”meant the ischemic stroke etiologic subtypes were classified according to the Trial of Org 10,172 in Acute Stroke Treatment criteria.

d Onset time was defined as time from stroke onset to recruitment/admission/diagnosis.

e Initial stroke therapy classified as anticoagulant therapy, antiplatelet therapy, thrombolysis, mechanical thrombectomy, or others.

f *Sample time was defined as time from stroke onset to take blood sample*.

### Overall Prognostic Analysis

**Figure 2:**
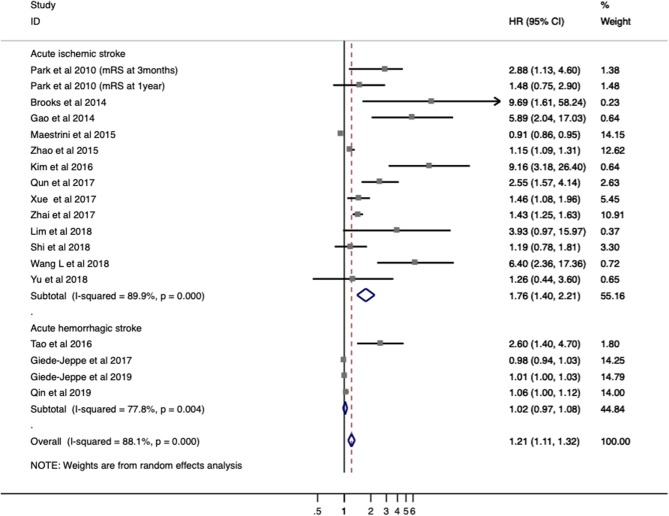
Meta-analysis of the association between NLR and modified Rankin Scale (mRS) functional outcome in patients. Results are presented as individual and pooled risk ratios (RRs) with 95% confidence intervals (CIs).

Seventeen studies with 5,858 patients provided ORs/RRs and 95% CIs for functional outcome. Unfavorable functional outcome was related to increased NLR in patients with stroke (ORs/RRs = 1.423; 95% CI = 1.218–1.662; *I*^2^ = 89.5%; *P* < 0.001; Figure 2). The negative effect of increased NLR levels was more pronounced in ischemic stroke (ORs/RRs = 1.609; 95% CI = 1.283–2.019) than in hemorrhagic stroke (ORs/RRs = 1.523; 95% CI = 0.590–3.931; Figure 2).

**Figure 3:**
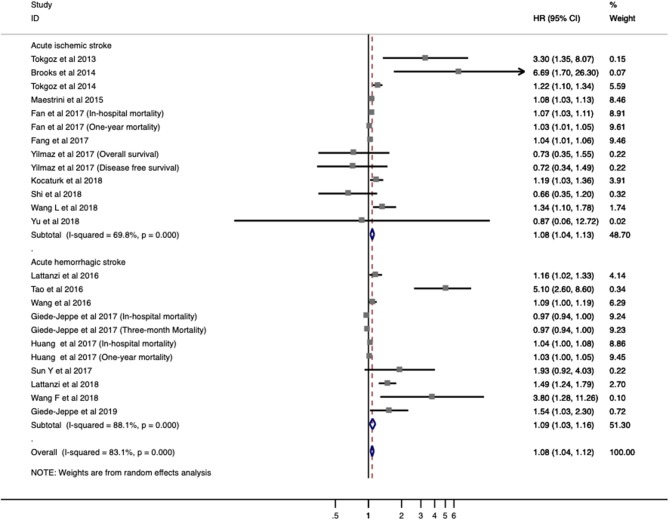
Meta-analysis of the association between NLR and mortality in patients. Results are presented as individual and pooled risk ratios (RRs) with 95% confidence intervals (CIs).

Twenty studies with 7,517 patients were analyzed for overall mortality. The pooled ORs/RRs of higher baseline NLR level was 1.067 (95% CI = 1.030–1.105; *I*^2^ = 83.9%; *P* < 0.001; Figure 3). Elevated NLR levels were associated with increased mortality in both ischemic stroke (ORs/RRs = 1.068; 95% CI = 1.027–1.111) and hemorrhagic stroke (ORs/RRs = 1.080; 95% CI = 1.018–1.146; Figure 3).

### Subgroup Prognostic Analysis in Ischemic Stroke

In subgroup analysis, functional outcome (Table 2) in ischemic stroke was according to four major factors, including assessment time, demographic factors (baseline NIHSS score, age, gender distribution, country), vascular risk factors (presence of hypertension, DM, hyperlipidemia, and current smoking) and methodological factors (onset time, sample time, cutoff value, definition of cutoff value, and ORs/RRs calculation). The poor prognostic effect of high NLR levels was only seen at 3 months (ORs/RRs = 1.851; 95% CI = 1.325–2.584; *I*^2^ = 91.7%; *P* < 0.001). Combined ORs/RRs remained significant in subgroups of male dominant populations and eastern countries. Poor functional outcomes were observed in non-elderly or elderly individuals with high NLR. Stroke severity with elevated NLR was not related to worse outcome. Furthermore, cohorts with higher presence of hypertension, DM, and current smoking were more likely to have unfavorable outcomes. With regard to methodological factors, we explored the relationship between the temporal profile of plasma NLR and functional outcomes. A poor prognosis was found in populations with continuously high NLR level at 48 h or long after stroke onset (ORs/RRs = 1.432; 95% CI = 1.266–1.619). The subgroup with higher plasma NLR on admission had the worst functional outcome (ORs/RRs = 3.291; 95% CI = 1.514–7.157). Cutoff values of plasma NLR varied among studies, and those with a cutoff value more than 4 were associated with worse ORs/RRs (ORs/RRs = 3.469; 95% CI = 1.904–6.320). ROC analysis was the most widely used method of assessment and had a relatively close relationship with worse outcomes (ORs/RRs = 2.306; 95% CI = 1.685–3.155). Finally, the estimated ORs/RRs from multivariate and univariate models were 2.076 (1.384–3.112) and 1.706 (1.200–2.426), respectively. In sensitivity analysis under “one study removed” model, the pooled ORs/RRs were significantly affected by exclusion of Maestrini et al. (28) (Supplementary Table 4). After removal of this study, heterogeneity decreased by 10% and the pooled ORs/RRs remained significant (ORs/RRs = 1.963; 95% CI = 1.526–2.524).

**Table 2:** Subgroup analyses of the associations between NLR and modified Rankin Scale (mRS) assessed functional outcome in ischemic stroke.

**Stratified analyses**	**No. of patients**	**No. of studies**	**Model**	**Pooled ORs/RRs (95% CI)**	***P*-value**	***P*_**D**_-value**	**Heterogeneity**	
							***I*^**2**^**	***P*_**H**_-value**
**Assessment time**						<0.001		
mRS at discharge	1,244	4	Random	2.548 (0.954–6.805)	0.062		85.1%	<0.001
mRS at 3 months	2,891	9	Random	1.851 (1.325–2.584)	<0.001		91.7%	<0.001
mRS at 1 year	371	1	Random	1.480 (0.753–2.910)	0.256			
**Demographic factors**								
Baseline NIHSS score^*^						0.010		
Mild	1,429	3	Random	2.211 (0.673–7.257)	0.191		86.4%	<0.001
Moderate to severe	1,477	4	Random	1.614 (0.874–2.978)	0.126		88.0%	<0.001
**Age**						<0.001		
<65	1,761	6	Random	1.508 (1.179–1.928)	0.001		80.9%	<0.001
≥65	1,899	5	Random	2.572 (1.062–6.229)	0.036		90.5%	<0.001
**Gender distribution**						<0.001		
Male dominant	3,018	9	Random	1.636 (1.307–2.047)	<0.001		75.6%	<0.001
Balanced	906	2	Random	0.930 (0.829–1.043)	0.216		83.2%	0.003
Female dominant	95	1	Random	6.400 (2.359–17.362)	<0.001		-	-
**Country**						<0.001		
Eastern	2,719	10	Random	1.932 (1.496–2.494)	<0.001		81.6%	<0.001
Western	1,416	3	Random	1.589 (0.580–4.354)	0.367		71.6%	0.030
**Vascular risk factors**								
**Presence of hypertension**						<0.001		
≥55% and <65%	1,935	3	Random	1.028 (0.826–1.280)	0.806		89.3%	<0.001
≥65% and <75%	1,018	5	Random	3.187 (1.969–5.157)	<0.001		52.3%	0.063
≥75%	971	3	Fixed	1.411 (1.254–1.588)	<0.001		0.0%	0.706
**Presence of diabetes mellitus**						<0.001		
<25%	2,450	5	Random	1.198 (0.948–1.514)	0.130		88.6%	<0.001
≥25%	1,474	6	Random	2.258 (1.531–3.331)	<0.001		74.1%	0.001
**Presence of hyperlipidemia**						0.002		
<25%	1,450	4	Random	2.463 (1.215–4.991)	0.012		83.4%	<0.001
≥25%	2,271	5	Random	1.216 (0.899–1.644)	0.204		91.3%	<0.001
**Presence of current smoking**						<0.001		
<35%	1,008	4	Random	1.826 (1.178–2.831)	0.007		57.2%	0.071
≥35%	2,070	6	Random	1.881 (1.298–2.728)	0.001		80.8%	<0.001
**Methodological factors**								
**Onset time**^**#**^						0.002		
Within 24 h	1,093	3	Random	1.821 (0.725–4.577)	0.202		90.6%	<0.001
Within 48 h	1,460	3	Random	1.485 (0.967–2.280)	0.071		56.7%	0.074
More than 48 h	599	2	Fixed	1.432 (1.266–1.619)	<0.001		0.0%	0.907
**Sample time**^**&**^						<0.001		
On admission	1,075	5	Random	3.291 (1.514–7.157)	0.003		78.5%	0.001
Within 24 h	694	3	Random	1.728 (1.157–2.580)	0.007		76.6%	0.014
Within 48 h	825	2	Random	1.858 (1.130–3.053)	0.015		18.6%	0.293
Within 72 h	635	1	Random	1.150 (1.046–1.264)	0.004		-	-
**Cutoff value**						<0.001		
<4	2,264	8	Random	1.941 (1.472–2.559)	<0.001		83.0%	<0.001
≥4	769	4	Fixed	3.469 (1.904–6.320)	<0.001		46.9%	0.130
**Definition of cutoff value**						0.267		
ROC curve analysis	2,095	9	Random	2.306 (1.685–3.155)	<0.001		84.4%	<0.001
Continuous variable	1,218	2	Fixed	0.913 (0.869–0.960)	<0.001		35.0%	0.215
**ORs/RRs calculation**^‡^						<0.001		
Multivariate	3,038	9	Random	2.076 (1.384–3.112)	<0.001		86.2%	<0.001
Univariate	1,097	4	Random	1.706 (1.200–2.426)	0.003		88.1%	<0.001

* Baseline stroke severity was assessed by the NIH Stroke Scale (NIHSS) and categorized into two groups with moderate to severe (score of 5 to 20 points) and mild (0 to 4 points). Only one study (13) use Scandinavian Stroke Scale (SSS) to evaluate stroke severity.

# Onset time was defined as time from stroke onset to recruitment/admission/diagnosis.

& Sample time was defined as time from stroke onset to take blood sample.

‡ *HRs were extracted from multivariate Cox proportional hazards models, univariate Cox proportional hazards models, or survival curve analysis*.

Table 3 demonstrates the relationship between NLR and mortality in ischemic stroke. Subgroups analysis was stratified by the four aforementioned major factors. Higher NLR levels were associated with both in-hospital mortality and long-term mortality. The elderly subgroup showed comparatively worse ORs/RRs (ORs/RRs = 1.131; 95% CI = 1.042–1.227). Pooled ORs/RRs from eastern countries was 1.046 (95% CI = 1.017–1.077), and that from western countries was 1.168 (95% CI = 1.018–1.339). ORs/RRs remained significant in subgroups stratified by all methodological factors.

**Table 3:** Subgroup analyses of the associations between NLR and mortality in ischemic stroke.

**Stratified analyses**	**No. of patients**	**No. of studies**	**Model**	**Pooled HR (95% CI)**	***P*-value**	***P*_**D**_-value**	**Heterogeneity**	
							***I*^**2**^**	***P*_**H**_-value**
**Assessment time**						<0.001		
**Short-term mortality**								
In-hospital mortality	2,642	4	Fixed	1.051 (1.029–1.072)	0.005		44.8%	0.142
30-day mortality	151	1	Random	1.220 (1.105–1.347)	<0.001		-	-
60-day mortality	255	1	Random	3.300 (1.350–8.068)	0.009		-	-
90-day mortality	1,437	4	Random	1.115 (0.913–1.362)	0.284		72.4%	0.013
Long-term mortality^*^	468	2	Random	1.030 (1.010–1.050)	0.003		0.0%	0.423
**Demographic factors**								
**Age**						<0.001		
<65	935	4	Random	1.052 (0.997–1.110)	0.065		55.4%	0.047
≥65	3,656	7	Random	1.131 (1.042–1.227)	0.003		77.3%	<0.001
**Gender distribution**						<0.001		
Male dominant	2,919	4	Fixed	1.039 (1.024–1.053)	<0.001		23.8%	0.263
Balanced	1,577	6	Random	1.170 (1.016–1.347)	0.030		70.8%	0.002
Female dominant	95	1	Random	1.339 (1.050–1.708)	0.019		-	-
**Country**						<0.001		
Eastern	2,560	4	Random	1.046 (1.017–1.077)	0.002		57.5%	0.052
Western	2,031	7	Random	1.168 (1.018–1.339)	0.027		66.0%	0.004
**Vascular risk factors**								
**Presence of hypertension**						<0.001		
<55%	257	2	Fixed	1.198 (1.088–1.321)	<0.001		45.3%	0.161
≥55% and <65%	1,658	4	Random	1.162 (0.986–1.370)	0.074		59.9%	0.058
≥65% and <75%	1,731	1	Random	1.040 (1.015–1.065)	0.001			
≥75%	734	2	Random	1.045 (1.001–1.090)	0.044		61.7%	0.074
**Presence of diabetes mellitus**						<0.001		
<25%	2,034	4	Fixed	1.043 (1.026–1.060)	<0.001		48.1%	0.103
≥25%	2,240	4	Random	1.167 (1.012–1.347)	0.034		83.9%	<0.001
**Presence of hyperlipidemia**						0.006		
<25%	619	3	Random	1.076 (1.013–1.143)	0.018		73.2%	0.005
≥25%	2,030	5	Random	1.125 (0.944–1.342)	0.189		60.2%	0.040
**Presence of current smoking**						0.214		
<35%	2,591	4	Random	1.167 (0.972–1.402)	0.098		81.0%	0.001
≥35%	372	1	Random	0.660 (0.356–1.222)	0.186			
**Methodological factors**								
**Onset-time**^**#**^						<0.001		
Within 24 h	1,355	4	Random	1.174 (1.043–1.321)	0.008		73.7%	0.010
Within 48 h	2,547	3	Fixed	1.039 (1.024–1.054)	<0.001		5.3%	0.367
**Sample time**^**&**^						0.001		
On admission	2,208	7	Random	1.083 (1.020–1.151)	0.010		75.3%	<0.001
Within 24 h	198	2	Fixed	1.222 (1.083–1.379)	0.396		0.0%	0.001
Within 48 h	2,185	2	Fixed	1.040 (1.015–1.065)	0.001		0.0%	0.896
**Cutoff value**						0.019		
<4	1,837	2	Fixed	1.039 (1.014–1.065)	0.002		0.0%	0.403
≥4	1,174	6	Random	1.308 (1.103–1.551)	0.002		56.0%	0.045
**Definition of cutoff value**						<0.001		
ROC curve analysis	2,302	6	Random	1.155 (1.012–1.319)	0.033		76.0%	<0.001
Continuous variable	1,580	3	Random	1.054 (1.017–1.092)	0.004		61.0%	0.053
Median	255	1	Random	3.300 (1.350–8.068)	0.009		-	−
**ORs/RRs calculation**^‡^						<0.001		
Multivariate	4,485	10	Random	1.085 (1.042–1.130)	<0.001		73.6%	<0.001
Univariate	106	1	Fixed	0.725 (0.429–1.225)	0.229		0.0%	0.979

* Long-term mortality was defined as hazard of death due to all causes or stroke within at least 1 year by the end of follow-up.

# Onset time was defined as time from stroke onset to recruitment/admission/diagnosis.

& Sample time was defined as time from stroke onset to take blood sample.

‡ *HRs were extracted from multivariate Cox proportional hazards models, univariate Cox proportional hazards models, or survival curve analysis*.

Patients had increased risk of hemorrhagic transformation after thrombolysis in ischemic stroke. Herein, we further evaluated the relationship between NLR levels and post-stroke complications. Higher NLR levels posed a higher risk of spontaneous ICH with thrombolysis (RRs = 1.290; 95% CI = 1.063–1.565; *I*^2^ = 87.1%; *P* < 0.001; Supplementary Figure 2).

### Subgroup Prognostic Analysis in Hemorrhagic Stroke

We only conducted subgroup analysis of mortality in hemorrhagic stroke (Table 4) as higher NLR level was not associated with poor functional outcomes in overall analysis (ORs/RRs = 1.523; 95% CI = 0.590–3.931). Elevated NLR was a negative prognostic factor in 90-day mortality. Increased mortality was observed in two types of hemorrhagic stroke, which are ICH (ORs/RRs = 1.090; 95% CI = 1.004–1.182) and SAH (ORs/RRs = 1.125; 95% CI = 1.002–1.263). Male dominant cohorts with high NLR had higher mortality rates (ORs/RRs = 1.944; 95% CI = 1.281–2.951). In addition, studies using ROC analysis to define optimal cutoff values were associated with negative pooled ORs/RRs. The estimated ORs/RRs of subgroups with cutoff values more than 4 was 1.547 (95% CI = 1.205–1.987).

**Table 4:** Subgroup analyses of the associations between NLR and mortality in hemorrhagic stroke.

**Stratified analyses**	**No. of patients**	**No. of studies**	**Model**	**Pooled HR (95%CI)**	***P*-value**	***P*_**D**_-value**	**Heterogeneity**	
							***I*^**2**^**	***P*_**H**_-value**
**Assessment time**						0.005		
In-hospital mortality	1,129	2	Random	1.002 (0.933–1.076)	0.961		88.3%	0.003
30-day mortality	613	3	Random	1.384 (0.985–1.945)	0.061		85.4%	0.001
90-day mortality	1,720	4	Random	1.489 (1.068–2.075)	0.019		92.2%	<0.001
One-year mortality	593	2	Random	1.196 (0.817–1.750)	0.358		73.9%	0.050
**Clinical characteristic**								
**Hemorrhagic stroke subtypes**						0.005		
ICH	1,997	6	Random	1.090 (1.004–1.182)	0.039		85.7%	<0.001
SAH	929	3	Random	1.125 (1.002–1.263)	0.046		90.4%	<0.001
**Hematoma size**						0.005		
<14 ml	737	3	Random	1.348 (1.067–1.702)	0.012		66.3%	0.052
≥14 ml	1,596	4	Random	1.061 (0.958–1.174)	0.255		90.5%	<0.001
**Presence of IVH**						0.024		
<25%	389	2	Random	2.027 (0.857–4.798)	0.108		63.8%	0.096
≥25%	1,191	2	Random	1.025 (0.916–1.146)	0.670		93.3%	<0.001
**Demographic factors**								
**Age**						0.011		
<65	962	3	Random	1.112 (0.989–1.250)	0.076		90.1%	<0.001
≥65	1,645	5	Random	1.080 (0.997–1.170)	0.059		87.1%	<0.001
**Gender distribution**						0.005		
Male dominant	1,301	5	Random	1.944 (1.281–2.951)	0.002		89.6%	<0.001
Balanced	855	1	Random	0.970 (0.950–0.991)	0.006		-	-
Female dominant	770	3	Random	1.051 (1.005–1.100)	0.030		55.8%	0.079
**Country**						0.005		
Eastern	1,367	5	Random	1.115 (1.011–1.229)	0.029		86.5%	<0.001
Western	1,559	4	Random	1.083 (0.993–1.181)	0.072		87.5%	<0.001
**Vascular risk factors**								
**Presence of hypertension**						0.005		
<55%	274	1	Fixed	1.033 (1.012–1.054)	0.002		0.0%	0.678
≥55% and <65%	655	2	Random	2.743 (0.849–8.863)	0.092		90.6%	0.001
≥65% and <75%	609	3	Random	1.214 (1.035–1.425)	0.017		78.1%	0.010
≥75%	1,388	3	Random	0.978 (0.923–1.037)	0.458		68.3%	0.024
**Presence of diabetes mellitus**						0.011		
<25%	1,752	7	Random	1.177 (1.072–1.291)	0.001		87.1%	<0.001
≥25%	855	1	Fixed	0.970 (0.950–0.991)	0.006		0.0%	0.740
**Presence of hyperlipidemia**						0.161		
<25%	177	1	Random	1.160 (1.016–1.325)	0.028			
≥25%	1,063	2	Random	1.034 (0.949–1.126)	0.449		90.4%	<0.001
**Presence of current smoking**						0.011		
<35%	1,928	5	Random	1.158 (1.033–1.297)	0.011		91.6%	<0.001
**Methodological factors**								
**Sample time**^****&****^						0.002		
On admission	1,398	3	Random	1.003 (0.950–1.059)	0.916		74.7%	0.008
Within 24 h	1,254	5	Random	1.965 (1.313–2.941)	0.001		86.4%	<0.001
**Cutoff value**						0.026		
<4	855	1	Fixed	0.970 (0.950–0.991)	0.006		0.0%	0.740
≥4	1,589	6	Random	1.626 (1.221–2.166)	0.001		85.2%	<0.001
**Definition of cutoff value**						0.005		
ROC curve analysis	1,445	6	Random	1.547 (1.205–1.987)	0.001		87.0%	<0.001
4th quartile	1,207	2	Random	0.972 (0.940–1.005)	0.091		41.8%	0.179
Continuous variable	274	1	Fixed	1.033 (1.012–1.054)	0.002		0.0%	0.678
**HR calculation**						0.011		
Multivariate	2,607	8	Random	1.080 (1.018–1.146)	0.011		88.7%	<0.001

ICH, intracerebral hemorrhage; SAH, subarachnoid hemorrhage; IVH, intraventricular hemorrhage.

*^#^Onset time was defined as time from stroke onset to recruitment/admission/diagnosis*.

& Sample time was defined as time from stroke onset to take blood sample.

*^‡^HRs were extracted from multivariate Cox proportional hazards models, univariate Cox proportional hazards models, or survival curve analysis*.

### Association of NLR and Risk of Ischemic Stroke

Seven articles reporting data from 35,367 subjects were estimated to evaluate the relationship between NLR and risk of ischemic stroke. We found a high statistically significant risk of ischemic stroke among individuals with elevated NLR levels (RRs = 2.074; 95% CI = 1.485–2.896; *I*^2^ = 93.6%; *P* < 0.001; Supplementary Figure 1). In addition, we explored the high heterogeneity by subgroup analysis stratified by ischemic stroke subtypes, demographic factors, vascular risk factors, and methodological factors (Supplementary Table 2). There was a negative relationship between risk of all subtypes of ischemic stroke and increased NLR levels. Risk of ischemic stroke was elevated when the population had high baseline NLR levels comorbid with higher presence of hypertension (RRs = 2.312; 95% CI = 1.238–4.321), DM (RRs = 1.942; 95% CI = 1.371–2.752), hyperlipidemia (RRs = 2.156; 95% CI = 1.204–3.861), and current smoking (RRs = 1.047; 95% CI = 1.011–1.084). Cutoff values of these articles were all <4. Majority of cutoff values were defined by ROC analysis. The combined RRs was 2.795 (95% CI = 1.685–4.636) in subgroup of ROC analysis.

### Publication Bias

We observed evidence of publication bias in studies providing functional outcomes in ischemic stroke (Supplementary Table 5) as well as mortality in hemorrhagic stroke (Supplementary Table 6) by Egger's test. Then, we applied the trim and fill method to address these problems. After the adjustment, the combined ORs/RRs of higher baseline NLR level were 1.088 (0.869–1.361) and 1.027 (0.957–1.102), respectively (Supplementary Tables 5, 6).

## Discussion

Literatures on NLR, as an inflammatory biomarker in cancer and cardiovascular disease, have grown exponentially over the past 5 years. Our meta-analysis evaluates the clinical significance of the NLR in stroke and adds a comprehensive systematic review to the cerebrovascular field. NLR is an easily acquired, non-invasive, and inexpensive marker, which can be used routinely to indicate systematic inflammatory status in clinical work. This is the first meta-analysis to comprehensively assess the clinical significance of NLR in both ischemic and hemorrhagic stroke under consistent methodology. In the setting of ischemic stroke, higher NLR levels were correlated with increased risk of stroke, unfavorable functional outcome at 3 months, and increased mortality, while in terms of hemorrhagic stroke (including SAH and ICH), elevated NLR levels only had deleterious effects on mortality.

The mechanism underlying the clinical significance of NLR on stroke is due to a central role of inflammation in all types of stroke from its initiation, progression of injury, and recovery (54–56). The inflammation cascade is initiated immediately by stagnant blood flow resulting from either ischemic or hemorrhagic lesion (5, 11). Release of proinflammatory mediators, such as TNF-α, IL-1, IL-6, and matrix metalproteinase-9 (MMP-9) from endothelium and brain parenchyma further potentiates tissue injury. Moreover, danger-/damage-associated molecular patterns (DAMP) are produced from injured and dying neurons. The main target of inflammation is the disruption of the brain–blood barrier (BBB) or neurovascular unit. Older animal studies have reported a biphasic behavior of BBB damage. However, recent human and animal studies indicate that BBB permeability remains elevated especially in the acute phase (6–48 h after stroke onset) due to the inflammatory cascade (57). Therefore, DAMP and proinflammatory mediators could gain access to the systemic circulation through the disrupted BBB or the cerebrospinal fluid (CSF) drainage system. Once in circulation, the systematic inflammatory response is potentiated. Among various types of peripheral inflammatory cells, neutrophils are the first to infiltrate the lesion (30 min to a few hours), peak earlier (24–72 h) and decrease rapidly with time (58). Locally, neutrophils participate in brain injury by exacerbating oxidative stress and BBB damage (59–61). The consequence of BBB breakdown is related to the many complications of stroke. Most commonly, pathologic cerebral edema results from increased BBB permeability and tends to develop within the first 24 to 48 h in AIS (62) or within the first 24 h in ICH (63). Breakdown of BBB is also associated with elevated risk of hemorrhagic transformation in AIS. Furthermore, inflammation is involved in the restoration of BBB function. After the production of proinflammatory factor peak and neutrophils in the acute/subacute phase (from onset to more than 48 h), neutrophil levels fall. This decrease during stroke recovery may help BBB integrity and be associated with good prognosis (64, 65). Therefore, the post-stroke inflammatory response has become a therapeutic target, as an adjacent treatment to reperfusion therapy using thrombolysis or intravascular clot removal (54, 66). Several drugs have been tested in randomized trials such as Fingolimod (67, 68), Natalizumab (69), Interleukin-1 receptor antagonist (IL-1ra) (70), and Minocycline (ACTRN12611001053910). The findings are anticipated to improve treatment options and clinical outcomes in of patients with acute stroke (59). Moreover, suppression of inflammation is also beneficial in models of cerebral hemorrhage (71). However, systemic immunosuppression follows after acute phase due to disturbed brain-immune interaction (4). Increased released glucocorticoids by the hypothalamic–pituitary axis and circulating epinephrine produced by the adrenal medulla or via the dense innervation by postganglionic sympathetic fibers of lymphoid organs are the major pathways to decrease lymphocyte counts, especially T cells and natural killer cells (3). Accordingly, infection is the most prevalent complication after stroke and contributes to the main cause of in-hospital death (66, 72, 73). This is consistent with our results that higher NLR levels were especially related to in-hospital mortality in ischemic stroke. Completing the cycle, NLR levels are elevated because of increased neutrophil counts and downsized lymphocyte counts in the post-stroke stage. Furthermore, elevated NLR levels had detrimental effects on prognosis due to secondary brain injury by neutrophil activation and increased risk of infection by lymphocyte suppression. Given the success of mechanical thrombectomy for large vessel occlusions, it would stand to reason that the NLR would fall in successful recanalization, given a lack of stagnating clot and reperfusion with less loss of BBB integrity. Abdalla et al. (74) reported their results with successful TICI 2b/3 recanalization and reported NLR fall 72 h post successful recanalization. The lower NLR level correlated directly with 90-day functional outcomes. Furthermore, an elevated neutrophil count was noted to be an independent predictor of poor outcome (>mRS3) at 90 days despite TICI 2b/3 recanalization by Bouisseau et al. (75) with higher infarct volumes. Thus, post-stroke NLR may serve as a marker of patients who may require hemicraniectomy for large infarcts despite recanalization. Recanalization of low-ASPECTS score, large-core strokes has been shown to decrease the rate of malignant transformation requiring hemicraniectomy, and reperfusion with decreasing NLR counts may be one explanation/marker (76). However, our meta-analysis was unable to evaluate the prognostic value of NLR in patients with a certain type of stroke treatment or with different infarct sizes due to insufficient data. We highly suggest that future studies could pay more attention on these issues.

Although ischemic and hemorrhagic stroke shared similar inflammatory reaction (6), we found that prognosis of hemorrhagic stroke was weakly predicted by NLR level in contrast with that of ischemic stroke. Higher NLR levels were associated with increased risk of ischemic stroke. These results may be due to prothrombotic state induced by inflammation responsible for ischemic stroke prodrome. During inflammation, leukocytes interact with platelets, endothelium, and coagulation factors and have been widely recognized as important contributors to facilitating hemostasis in physiological and pathological conditions. This mechanism can also explain similar results in other clinical articles. For example, leukocytosis does not independently predict poor ICH prognosis when controlling for other outcome determinants including age, baseline hematoma volume, and admission Glasgow Coma Scale (77). Similarly, as hematoma expansion is related to poor outcome in hemorrhagic stroke (78), the inverse relationship between neutrophil counts and risk of hematoma expansion might relate to better prognosis (79). However, interestingly, elevated baseline NLR levels were also correlated with higher risk of hemorrhagic transformation after thrombolysis in ischemic stroke. This may be associated with antithrombotic effect of thrombolysis vs. a leaky BBB integrity. Thus, further experimental and clinical studies are needed to evaluate the predicting role of NLR in patients after thrombolysis.

In subgroup analysis, we found that prognostic value of NLR in stroke remained significant in subgroups of more than 65 years, male dominant composition, and patients from eastern countries, which are consistent with prior studies (14–16). Furthermore, as thromboembolism is the most common cause of ischemic stroke, we evaluated the vascular risk factors among the included studies. Cohorts with higher presence of hypertension (>65%), DM (>25%), and current smoking (>35%) tended to have more unfavorable functional outcomes in ischemic stroke. Cutoff values varied between studies due to different definitive methods, blood sampling time, and capacity of immune system (16). A higher cutoff value (>4) indicated poorer prognosis in stroke. In addition, we observed that cutoff values defined by ROC curves were more likely to predict poor clinical outcomes. Thus, future studies are suggested to determine their specific cutoff values by ROC curves. Temporal dynamics of neutrophil and lymphocyte counts have been described in previous studies (3, 80). Therefore, we conducted subgroup analysis stratified by onset time and sample time. Shorter time from stroke onset to admission (within 24 h), and quicker procurement of the blood sample (within 72 h) were beneficial to record the NLR level at early stages of stroke-induced inflammation and helped predict negative prognosis.

In this meta-analysis, baseline NLR was identified as a robust predictor of ischemic stroke occurrence and prognosis. However, there are several limitations. Firstly, considerable heterogeneity was found when combined ORs/RRs for functional outcomes and mortality were assessed. In the setting of ischemic stroke, heterogeneity was tremendously decreased to <50% after subgroup analysis of mortality assessment time, age, gender, country, and vascular risk factor. We further conducted sensitivity analysis of studies reporting functional outcomes in ischemic stroke and the outcomes had no significant change after excluding a single study. Secondly, publication bias existed in studies providing functional outcomes in ischemic stroke as well as mortality in hemorrhagic stroke. The negative effect of higher NLR was slightly reduced after adjustment of publication bias by the trim and fill method. Therefore, future studies are encouraged to publish null results to avoid overestimation of clinical significance of NLR. We excluded studies if their populations were complicated with autoimmune disorders or systematic inflammatory disorders to avoid the influence of chronic inflammatory status on NLR value (3, 81). However, it is also worth evaluating the clinical significance of NLR in patients with inflammatory conditions prior to enrollment as stroke can also manifest as a complication of inflammation. Finally, we observed that our included studies only reported the negative effect of high baseline NLR on all-cause mortality. As NLR is a reflection of inflammatory status, we highly suggest that future studies could specify the cause of death related to inflammation in post-stroke patients, such as infection-related death.

## Conclusions

Baseline NLR level is a promising predictor of ischemic or hemorrhagic stroke prognosis. Elevated NLR is also associated with high risk of ischemic stroke occurrence. Shorter time from stroke onset to admission (within 24 h) and timely procurement of blood samples may help to reflect the early inflammatory response of neutrophils and lymphocytes, which may predict clinical outcomes. Cutoff values of more than 4 may be related to worse prognosis. Future studies are needed to improve the aforementioned limitations and demonstrate the underlying mechanisms of our work here.

## Data Availability Statement

The raw data supporting the conclusions of this manuscript will be made available by the authors, without undue reservation, to any qualified researcher.

## Ethics Statement

The corresponding local ethics committee approved this study and all participants provided informed consent.

## Author Contributions

RM: manuscript drafting and revision, and study concept and design. S-YS: manuscript drafting and revision, study concept and design, collection, assembly, and interpretation of the data. X-XZ: collection, assembly, and interpretation of the data. RM, S-YS, X-XZ, CH, RK, and YH: manuscript writing and final approval of manuscript. GR and YD deeply edited the revised version and contributed critical revision.

### Conflict of Interest

The authors declare that the research was conducted in the absence of any commercial or financial relationships that could be construed as a potential conflict of interest.

## Abbreviations

NLR: neutrophil-to-lymphocyte ratio
AIS: acute ischemic stroke
mRS: modified Rankin Scale
NIHSS: National Institutes of Health Stroke Scale
GCS: Glasgow Coma Score
OR: odds ratio
RR: risk ratio
SAH: subarachnoid hemorrhage
ICH: intra-cerebral hemorrhage
IL: interleukin
CCL: chemokine (C-C motif) ligand 2
MMP-9: matrix metalloproteinase-9
PMC: PubMed Central
CI: confidence interval
LAA: large artery atherosclerosis
CE: cardioembolism
SVC: small vessel occlusion
CVST: cerebral venous sinus thrombosis
DM: diabetes mellitus
ROC: region under the curve
DAMP: danger-/damage-associated molecular patterns
BBB: brain–blood barrier
CSF: cerebrospinal fluid
ROS: reactive oxygen species
RNS: reactive nitrogen species
TICI: Thrombolysis in cerebral infarction
ASPECTS: Alberta Stroke Program Early CT Score.
