# Supplementary material for: Clinical Significance of Baseline Neutrophil-to-Lymphocyte Ratio in Patients With Ischemic Stroke or Hemorrhagic Stroke: An Updated Meta-Analysis
Source: Front Neurol. 2019 Oct 4;10:1032. doi: 10.3389/fneur.2019.01032 (PMC6787274; doi:10.3389/fneur.2019.01032)
Supplement: Supplementary file 1 [file Data_Sheet_1.pdf]

Supplementary Table. 1 Search Strategies

Search included: PUBMED, EMBASE: search date was from the inception through April 2019

1) Pubmed search strategy

1. "stroke"[Mesh]
2. Brain Ischemia [Title/Abstract]
3. Brain infarction [Title/Abstract]
4. cerebral infarction [Title/Abstract]
5. intracerebral hemorrhage [Title/Abstract]
6. intracranial hemorrhage [Title/Abstract]
7. 1 OR 2 OR 3 OR 4 OR 5 OR 6
8. "neutrophil"[MeSH Terms]
9. "lymphocyte"[MeSH Terms]
10. "neutrophil to lymphocyte ratio"[All Fields]
11. "NLR"[All Fields]
12. 8 OR 9 OR 10 OR 11
13. "Survival"[Mesh]
14. "Mortality"[Mesh]
15. "Prognosis"[Mesh]
16. Prognos*[Title/Abstract]
17. outcome*[Title/Abstract]
18. survival [Title/Abstract]
19. mortality [Title/Abstract]
20. predict*[Title/Abstract]
21. 13 OR 14 OR 15 OR 16 OR 17 OR 18 OR 19 OR 20
22. 7 AND 12 AND 21

2) Embase search strategy

1. 'stroke'/exp
2. Brain Ischemia:ab,ti
3. Brain infarction:ab,ti
4. cerebral infarction:ab,ti
5. intracerebral hemorrhage:ab,ti
6. intracranial hemorrhage:ab,ti
7. 1 OR 2 OR 3 OR 4 OR 5 OR 6
8. neutrophil to lymphocyte ratio'/exp
9. neutrophil to lymphocyte ratio
10. neutrophil to lymphocyte ratio: ab, ti
11. neutrophil: ab, ti
12. lymphocyte: ab, ti
13. NLR: ab, ti
14. 8 OR 9 OR 10 OR 11 OR 12 OR 13
15. 'prognosis'/exp
16. 'survival'/exp
17. 'mortality'/exp
18. prognos*: ab, ti
19. outcome*: ab, ti
20. survival: ab, ti
21. treatment: ab, ti
22. mortality': ab, ti
23. recurren*: ab, ti
24. predict*: ab, ti
25. 15 OR 16 OR 17 OR 18 OR 19 OR 20 OR 21 OR 22 OR 23 OR 24
26. 7 AND 14 AND 25

Supplementary Table. 2 Subgroup analyses of the associations between NLR and risk of ischemic stroke.

**Stratified analyses**	**No. of patient**	**No. of**	**Model**	**Pooled HR (95%CI)**	**P value**	**P_D value**	**Heterogeneity**	
							**I^2**	**P_H value**
**Clinical characteristic**								
**Ischemic stroke subtypes***						<0.001		
Mixed	2083	3	random	1.856 (1.169-2.948)	0.009		94.4%	<0.001
Atherosclerotic stroke	292	2	fixed	7.985 (4.070-15.667)	<0.001		0.0%	0.874
Cardioembolic stroke	32912	1	random	1.520 (1.261-1.832)	<0.001			
Cerebral venous sinus thrombosis (CVST)	80	1	random	1.442 (1.086-1.915)	0.011			
**Demographic factors**								
**Age**						<0.001		
< 65	470	4	random	2.542 (1.482-4.357)	0.001		89.7%	<0.001
≥ 65	34897	3	random	1.872 (1.109-3.160)	0.019		95.4%	<0.001
**Gender distribution**						<0.001		
Female dominant	2315	4	random	1.842 (1.229-2.760)	0.003		91.8%	<0.001
Balanced	60	1	random	46.820 (14.429-151.927)	<0.001			
Male dominant	32992	2	fixed	1.496 (1.280-1.748)	<0.001		0.0%	0.761
**Country**						<0.001		
Eastern	2083	3	random	1.856 (1.169-2.948)	0.009		94.4%	<0.001
Western	33284	4	random	2.578 (1.439-4.617)	0.001		86.7%	<0.001
**Vascular risk factors**								
**Presence of hypertension**						<0.001		
≥ 65% and <75%	34703	3	random	2.312 (1.238-4.321)	0.009		96.4%	<0.001
≥ 75%	546	2	random	2.156 (1.204-3.861)	0.010		89.6%	<0.001
**Presence of diabetes mellitus**						<0.001		
≥ 25%	35189	4	random	1.942 (1.371-2.752)	<0.001		94.1%	<0.001
**Presence of hyperlipidemia**						0.010		
≥ 25%	546	2	random	2.156 (1.204-3.861)	0.010		89.6%	<0.001
**Presence of current smoking**						0.002		
< 35%	584	3	random	4.145 (0.975-17.621)	0.054		91.7%	<0.001
≥ 35%	2023	2	random	1.047 (1.011-1.084)	0.010		74.5%	0.020
**Methodological factors**								
**Sample-time**&						0.001		
on admission	33030	3	random	1.600 (1.150-2.226)	0.005		60.7%	0.078
within 24 hours	292	1	random	1.499 (1.161-1.935)	0.002			
more than 24 hours	2277	3	random	1.797 (1.065-3.034)	0.028		94.1%	<0.001
**Cut-off value**						<0.001		
< 4	35367	7	random	1.906 (1.427-2.546)	<0.001		93.9%	<0.001
**Definition of cut-off value**						<0.001		
ROC curve analysis	2455	6	random	2.795 (1.685-4.636)	<0.001		94.2%	<0.001
4th quartile	32912	1	random	1.520 (1.261-1.832)	<0.001			
continuous variable	33204	2	random	1.235 (0.891-1.713)	0.205		84.8%	0.010
**HR calculation**‡						<0.001		
Multivariate	33576	5	random	1.802 (1.349-2.406)	<0.001		79.5%	<0.001
Univariate	1791	2	random	6.655 (0.160-277.341)	0.319		97.5%	<0.001

***: This system of categorizing stroke was based on the multicenter Trial of Org 10172 in Acute Stroke Treatment (TOAST).**

^: Risk of ischemic stroke was defined as atherosclerotic or lacunar or cardioembolic or cryptogenic stroke.

#: Onset-time was defined as time from stroke onset to recruitment/admission/diagnosis.

&: Sample-time was defined as time from stroke onset to take blood sample.

‡: HRs were extracted from multivariate cox proportional hazards models, univariate cox proportional hazards models or survival curve analysis.

Supplementary Table. 3 Quality assessment of eligible studies

No.	Authors (Ref.) *	Representativeness of population	Non-exposed cohort	Ascertainment of exposure	Outcome present at start of study	Appropriate confounding measurement and account	Sufficient measurement of outcomes	Completeness of follow-up
1	Park et al 2010	1	0	0	2	2	2	0
2	Tokgoz et al 2013	2	0	0	2	2	2	1
3	Akil et al 2014	1	0	0	2	2	2	2
4	Brooks et al 2014	1	0	0	2	1	2	1
5	Gao et al 2014	1	0	0	2	1	2	0
6	Tokgoz et al 2014	2	0	0	1	2	2	1
7	Maestrini et al 2015	2	0	0	2	2	2	1
8	Saliba et al 2015	1	0	0	2	1	2	2
9	Zhao et al 2015	2	0	0	2	2	2	0
10	Guo et al 2016	2	0	0	2	2	2	0
11	Kim et al 2016	1	0	0	2	1	2	0
12	Köklü et al 2016	2	0	0	2	2	2	2
13	Lattanzi et al 2016	2	0	0	2	2	2	0
14	Wang et al 2016	1	0	0	2	1	2	0
15	Tao et al 2016	1	0	0	2	1	2	0
16	Akboga et al 2017	1	0	0	2	1	2	0
17	Fan et al 2017	1	0	0	2	2	2	2
18	Fang et al 2017	1	0	0	2	1	2	0
19	Giede-Jeppe et al 2017	1	0	0	2	1	2	0
20	Huang et al 2017	1	0	0	2	1	2	0
21	Lattanzi et al 2017	2	0	0	2	2	2	0
22	Qun et al 2017	1	0	0	2	1	2	1
23	Sun Y et al 2017	1	0	0	2	1	2	1
24	Tao et al 2017	1	0	0	2	1	2	1
25	Xue et al 2017	2	0	0	2	2	2	2
26	Yilmaz et al 2017	1	0	0	2	1	2	0
27	Zhai et al 2017	1	0	0	2	2	2	1
28	Lattanzi et al 2018	2	0	0	2	2	2	1
29	Wang F et al 2018	1	0	0	2	1	2	0
30	Nam et al 2018	2	0	0	2	2	2	0
31	Shi et al 2018	1	0	0	2	1	2	0
32	Yu et al 2018	2	0	0	2	2	2	0
33	Kocaturk et al 2018	2	0	0	2	2	2	1
34	Lim et al 2018	2	0	0	2	2	2	1
35	Wang L et al 2018	1	1	0	2	1	2	2
36	Giede-Jeppe et al 2019	2	0	0	2	1	2	2
37	Qin et al 2019	2	0	0	2	2	2	0

Adequate assessment included 1) representativeness of population: “source population clearly defined” and “study population described” or “study population represents source population or population of interest”; 2) completeness of follow-up: “completeness of follow-up adequate”; 3) non exposed cohort: Drawn from the same community as the exposed cohort; 4) sufficient measurement of outcomes: “outcome measured appropriately”; 5) appropriate confounding measurement and account: “confounders defined and measured” and “confounding accounted for”; and 6) outcome of interest was not present at start of study

*References as described in manuscript

Supplementary Table. 4 Sensitivity analysis using a "one-study removed" model for functional outcome in ischemic stroke.

Sensitivity analysis	Heterogeneity test (I^2)	Pooled HR (95%CI)
All studies	89.9%	1.756 (1.395, 2.209)
Excluding Maestrini et al 2015	79.7%	1.963 (1.526, 2.524)

Supplementary Table. 5 Publication bias assessment with different tests for mortality and functional outcome in ischemic stroke.

Publication bias	Begg's *P* value	Egger's *P* value	T&F(Fill) method analysis		Model
			Before	After	
Mortality subset	0.246	0.096	-	-	-
Functional outcome subset	0.743	0.000	1.756 (1.395, 2.209)	1.088 (0.869, 1.361)	random

Abbreviations: CI= confidence interval; Fill=number of studies added by trim and fill method; het= heterogeneity; HR=hazard ratio; T&F=result of trimmed and filled analysis, using assumption of random effects.

Supplementary Table. 6 Publication bias assessment with different tests for mortality and functional outcome in hemorrhagic stroke.

Publication bias	Begg's *P* value	Egger's *P* value	T&F(Fill) method analysis		Model
			Before	After	
Mortality subset	0.026	0.003	1.089 (1.026, 1.157)	1.027 (0.957, 1.102)	random
Functional outcome subset	0.308	0.370	-	-	-

Abbreviations: CI= confidence interval; Fill=number of studies added by trim and fill method; het= heterogeneity; HR=hazard ratio; T&F=result of trimmed and filled analysis, using assumption of random effects.

Supplementary figures

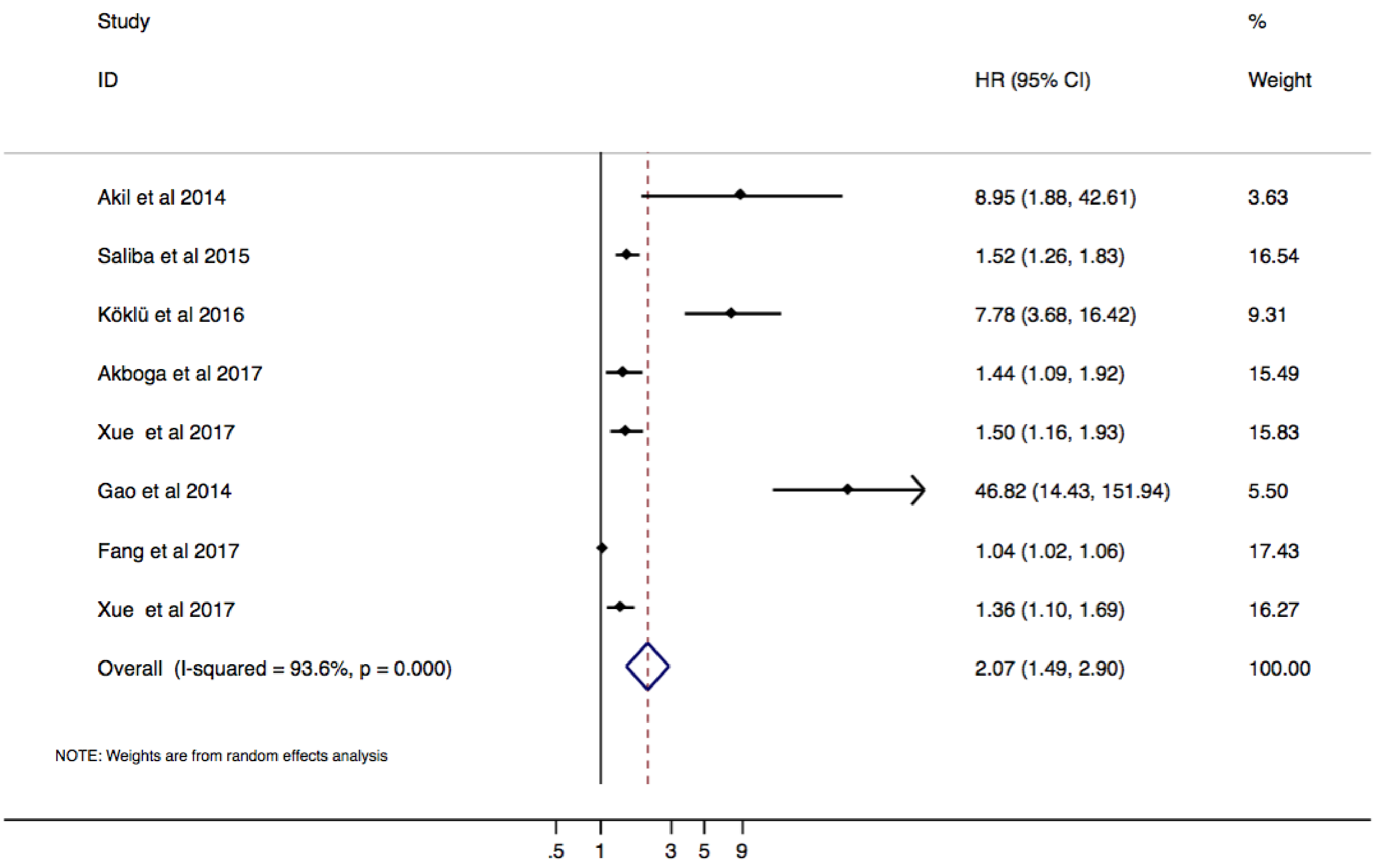

Fig. 1. Meta-analysis of the association between NLR and ischemic stroke incidence in patients. Results are presented as individual and pooled risk ratios (RRs) with 95% confidence intervals (CIs).

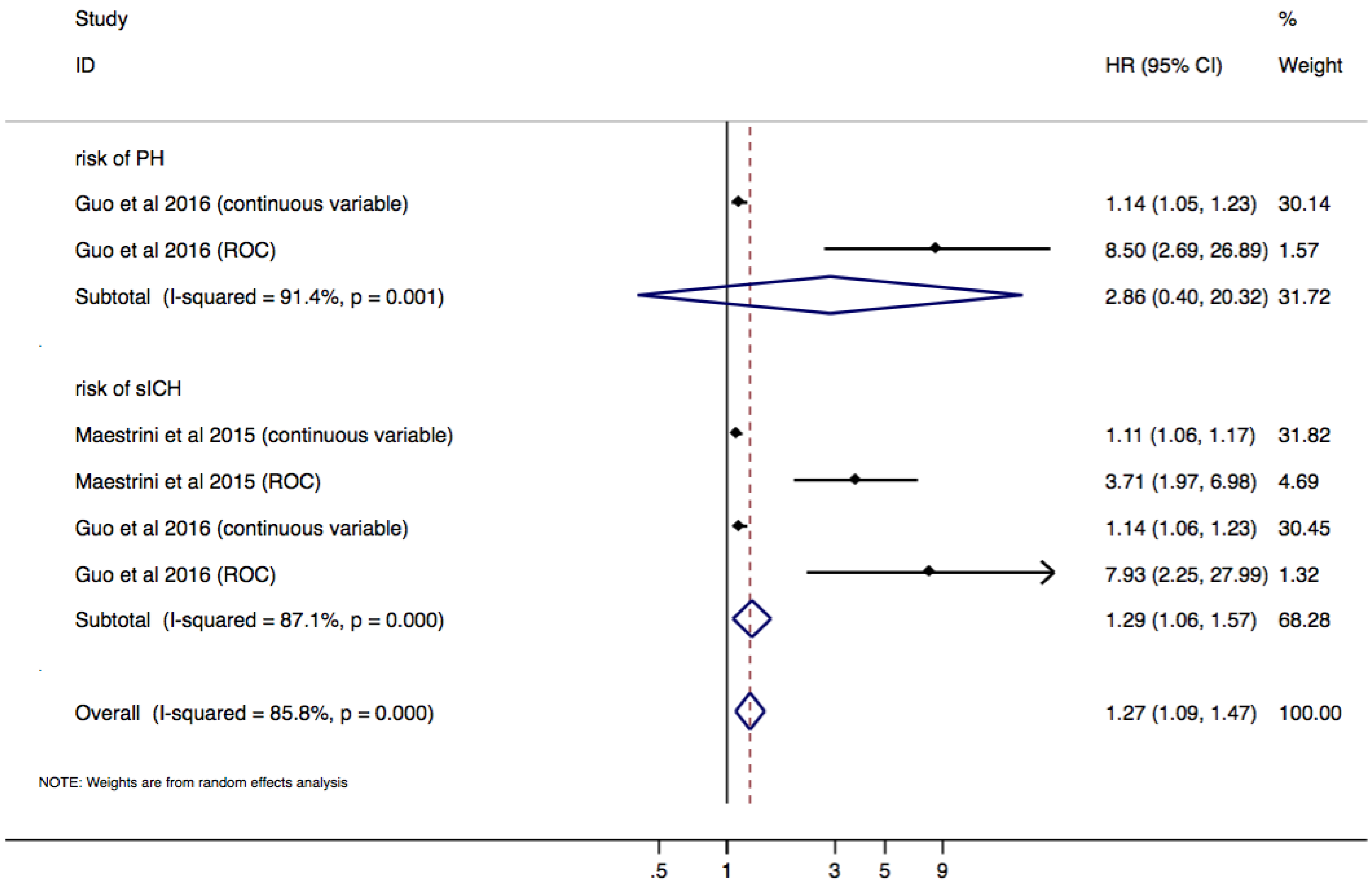

Fig. 2. Meta-analysis of the association between NLR and ischemic stroke complication incidence in patients. Results are presented as individual and pooled risk ratios (RRs) with 95% confidence intervals (CIs).

PRISMA checklist

Section/topic	#	Checklist item	Reported on page #
**TITLE**			
Title	1	Identify the report as a systematic review, meta-analysis, or both.	Page 1
**ABSTRACT**			
Structured summary	2	Provide a structured summary including, as applicable: background; objectives; data sources; study eligibility criteria, participants, and interventions; study appraisal and synthesis methods; results; limitations; conclusions and implications of key findings; systematic review registration number.	Page 1
**INTRODUCTION**			
Rationale	3	Describe the rationale for the review in the context of what is already known.	Page 3
Objectives	4	Provide an explicit statement of questions being addressed with reference to participants, interventions, comparisons, outcomes, and study design (PICOS).	Page 5
**METHODS**			
Protocol and registration	5	Indicate if a review protocol exists, if and where it can be accessed (e.g., Web address), and, if available, provide registration information including registration number.	Page 4
Eligibility criteria	6	Specify study characteristics (e.g., PICOS, length of follow-up) and report characteristics (e.g., years considered, language, publication status) used as criteria for eligibility, giving rationale.	Page 4
Information sources	7	Describe all information sources (e.g., databases with dates of coverage, contact with study authors to identify additional studies) in the search and date last searched.	Page 4
Search	8	Present full electronic search strategy for at least one database, including any limits used, such that it could be repeated.	Page 4
Study selection	9	State the process for selecting studies (i.e., screening, eligibility, included in systematic review, and, if applicable, included in the meta-analysis).	Page 4 &5
Data collection process	10	Describe method of data extraction from reports (e.g., piloted forms, independently, in duplicate) and any processes for obtaining and confirming data from investigators.	Page 5
Data items	11	List and define all variables for which data were sought (e.g., PICOS, funding sources) and any assumptions and simplifications made.	Page 5
Risk of bias in individual studies	12	Describe methods used for assessing risk of bias of individual studies (including specification of whether this was done at the study or outcome level), and how this information is to be used in any data synthesis.	Page 6
Summary measures	13	State the principal summary measures (e.g., risk ratio, difference in means).	Page 6
Synthesis of results	14	Describe the methods of handling data and combining results of studies, if done, including measures of consistency (e.g., I^2) for each meta-analysis.	Page 6
